# Buzzing towards Resilience: Investigating the Spatial Alignment of the Desert Pallid Bee, *Centris pallida*, and Its Host Plants in Response to Climate Change

**DOI:** 10.3390/insects15100793

**Published:** 2024-10-11

**Authors:** Terese Maxine P. Cruz, Stephen L. Buchmann, Kathleen L. Prudic

**Affiliations:** 1 School of Natural Resources and the Environment, University of Arizona, Tucson, AZ 85721, USA; 2 Department of Ecology and Evolutionary Biology, University of Arizona, Tucson, AZ 85721, USA; buchmann.stephen@gmail.com; 3 Department of Entomology, University of Arizona, Tucson, AZ 85721, USA; 4 Arizona Institute for Resilience, University of Arizona, Tucson, AZ 85721, USA; 5 BIO5 Institute, University of Arizona, Tucson, AZ 85721, USA

**Keywords:** habitat suitability predictions, maximum entropy model, native pollinator, species distribution models

## Abstract

**Simple Summary:**

Insect declines have been linked to rising temperatures and aridification due to global climate change. These declines impact ecosystems, as many flowering plants rely on insect pollinators like bees for reproduction. However, desert organisms, which are well-adapted to hot and arid conditions, may respond differently to environmental changes compared to those in non-desert environments. In this study, we estimate the overlapping suitable habitat for the desert pallid bee (*Centris pallida*) and three of its host plants—the desert ironwood (*Olneya tesota*), blue palo verde (*Parkinsonia florida*), and yellow palo verde (*Parkinsonia microphylla*)—under current and forecasted climate conditions using maximum entropy modeling (MaxEnt). MaxEnt creates predictions of where a species is most likely to reside in novel or known areas based on the environmental variables at locations of observed occurrences. We found that *C. pallida* and its host plants may exhibit resilience to warming temperatures from a moderate increase in greenhouse gasses as projected by climate models, resulting in a slight northern expansion of suitable habitats shifted to higher average altitudes where all four species may exist. This study might serve as a reference for future modeling studies and insight into the resilience of desert-dwelling pollinators.

**Abstract:**

Wild bees are vital for the pollination of native plants and crops, providing essential ecosystem services. Climate change is known to impact biodiversity and species distributions, but insects adapted to desert ecosystems may exhibit unique physiological, behavioral, and evolutionary responses. The desert pallid bee (*C. pallida*), a solitary bee native to the arid southwestern United States and northern Mexico, primarily forages on yellow palo verde (*P. microphylla*), blue palo verde (*P. florida*), and desert ironwood (*O. tesota*). This study used MaxEnt to estimate the current and projected geographical overlap of suitable habitats for *C. pallida* and its host plants. Here, we used MaxEnt to estimate the current and forecasted overlapping geographically suitable habitat of *C. pallida* with all three host plants. We forecasted potential environmentally suitable areas for each species to the year 2040 using the current distribution model and climate projections with moderate CO_2_ levels. We found a continued spatial alignment in the suitable area of the bee and its host plants with a 70% increase in the range overlap area, though shifted to higher average altitudes and a slight northern expansion. These findings may provide insight to stakeholders on the conservation needs of desert-dwelling pollinators.

## 1. Introduction

Insect pollinators are crucial for ecosystem health and economic activities, but their survival is increasingly threatened by climate change, which brings rising temperatures and increased aridity (e.g., [1,2,3]). Despite efforts towards climate mitigation and adaptation plans, organisms continue to face high temperature extremes alongside habitat fragmentation due to urbanization and agriculture [4]. These environmental changes disrupt the alignment between pollinators and their host plants, altering ecosystem structures and pollination services (e.g., [4,5,6,7]).

Wild bees are often more effective pollinators than domesticated honey bees (Apidae: *Apis mellifera* (Linnaeus, 1758)) with respect to native flora [8,9]. Monitoring the presence and absence of wild bees and their native plant hosts is a major factor in preserving local biodiversity as temperatures rise [10], but climate change-driven declines in native bee diversity have been observed most prominently in temperate and tropical ecosystems [11,12]. Some species with higher tolerances to heat and aridity fared better than those that were less tolerant [11,12,13], warranting the question of whether desert-adapted species may respond differently to a warming climate as they possess adaptations to persist through continued desertification [14,15].

The interplay between spatial distributions and climate is crucial for understanding wild bee ecology. Many pollinators depend on their host plants for nectar and pollen, and the availability of flowering desert plants—often drought-tolerant—can influence bee abundance and diversity [16]. The presence of certain plant species influences the abundance and diversity of bees in the area, an outcome often considered in conservation and may play a role in supporting the persistence of plant-feeding organisms [17,18]. Desert plants themselves appear to be persistent and even increase in abundance with climatic warming and aridification [19], and some desert bee populations are found to maintain a constant population density [20]. For example, a study on an arid-zone bee species (Apidae: *Exoneurella tridentata* (Houston, 1976)) and its host plants in Australia suggests that desert bees might be less vulnerable to climate change than those in subtropical and tropical regions [21]. However, future changes in climate could still exceed physiological limits and negatively affect individual survival, population persistence, and habitat suitability [22,23,24,25]. Some species might mitigate the effects of climate change on themselves by relocating to new habitats, changing in latitude, or climbing in elevation [26,27,28,29]. Estimating future species distributions under different climate change scenarios is helpful for identifying locations for protected areas as spatial distributions change, for informing decision-making on habitat enhancement and restoration activities, and for suggesting areas where surveys could be performed to gather more information on population trends (e.g., [30,31]).

Assessing population persistence may require a more holistic approach, considering not only species adaptations but also the condition of dependent species and related habitats (e.g., [32,33]). Along with population-level studies evaluating declines, modeling species distributions under varying climate scenarios can help identify potential locations for conservation and guide future research [34,35]. One method for estimating species distributions utilizes the maximum entropy model (MaxEnt). MaxEnt is a species distribution model (SDM) based on machine learning that is frequently used in ecological and conservation studies (e.g., [36,37,38]). All species distribution models are continually evolving to better manage sampling bias and improve predictive performance, so it is important to use the results thoughtfully for conservation planning. However, SDMs continue to offer valuable insight and assist in management decision-making (e.g., [37,39,40,41]). Applying SDMs to desert ecosystems—known for their diverse and abundant wild bee populations [42]—can enhance our understanding of regions potentially most affected by ongoing climate change.

Our study investigates organisms native to the arid deserts of northern Mexico and the southwest United States of America (U.S.), regions historically for deficits in precipitation and extreme temperatures [43,44]. These characteristics make it a region with the potential to become no longer physiologically suitable for either insects or their plant hosts in future years. We focus on the desert pallid bee (Apidae: *Centris pallida* (Fox, 1899)), a solitary species that adjusts its behavior to avoid extreme temperatures, such as seeking shade or cooler microhabitats [45,46,47,48]. This bee, crucial for pollinating desert plants like palo verde trees, also aids soil health through its burrowing [45,49]. Here, we aim to predict the geographically suitable habitat for *C. pallida* and its major host plants—the desert ironwood (Fabaceae: *Olneya tesota* (A. Gray, 1854)), blue palo verde (Fabaceae: *Parkinsonia florida* (Benth. ex A. Gray)) and yellow palo verde (Fabaceae: *Parkinsonia microphylla* (Torr.)) (also known as the little-leaved or foothills palo verde)—under current and projected climate conditions. We explore whether this desert pollinator and its host plants are likely to be threatened in the future with respect to a warming climate.

## 2. Materials and Methods

### 2.1. Study System

We focused on observations in the desert regions of the southwestern United States of America and northwestern Mexico, which includes the Mojave and Sonoran deserts, located between latitudes 20° N and 40° N and longitudes 105° W and 125° W. Both regions accumulate less than 500 mm of precipitation a year depending on the location, with mountainous regions receiving the upper limit. The Mojave receives a lower amount relative to the Sonoran since its precipitation is derived primarily from winter rains, while the Sonoran Desert receives precipitation from winter and summer rains. The two deserts are often regarded as the driest (Mojave) and most subtropical (Sonoran) deserts in North America, offering a range of ecosystems within the study area. Average temperatures range around a low of 50 °F (10 °C) during the cooler months to a high of 104 °F (40 °C), sometimes reaching 118 °F (48 °C), from June to August [50,51]. Differences abound between the two deserts related to geology and soil types in addition to precipitation [50,52].

**Figure 1:**
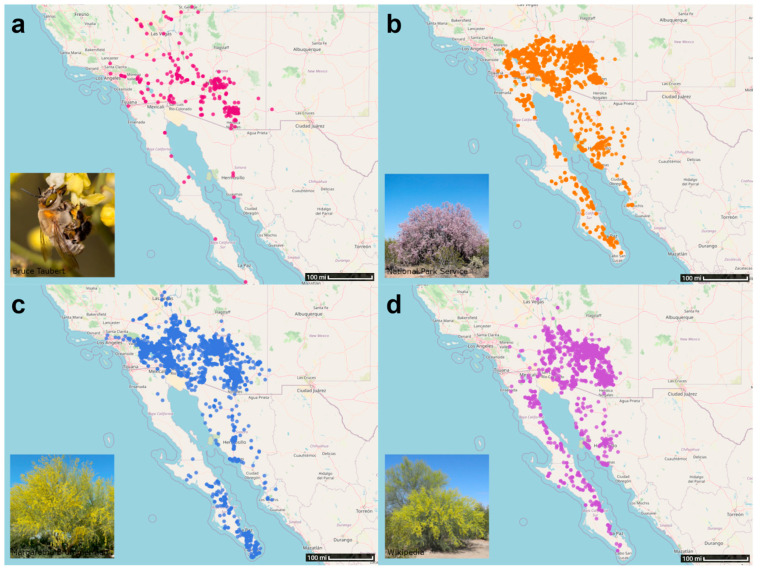
Occurrence maps showing observations of our focal species. Panels show (**a**) *C. pallida*, (**b**) *O. tesota*, (**c**) *P. florida*, and (**d**) *P. microphylla*. An inset photograph of each species is presented in the bottom left corner of each panel.

Our focal species included the desert pallid bee (*C. pallida*), desert ironwood (*O. tesota*), blue palo verde (*P. florida*) and yellow palo verde (*P. microphylla*) (Figure 1). *C. pallida* is a solitary bee that nests underground, constructing brood cells 4 to 11 cm deep in sandy to gravelly soil [Sabino and Buchmann, unpublished]. It feeds on nectar and pollen from *O. tesota*, *P. florida*, and *P. microphylla*, which supports both adult and larval survival [45,49]. Notably, this bee shows a strong preference for pollen from palo verde trees [49]. All study species can withstand high temperatures and tend to reside at elevations below 4000 feet (1219 m), though some observations for all organisms have been observed at greater elevations (Table S1). The host plants are desert-adapted, requiring little to no water after establishment, and are hardy to average winter minimums of 15–30 °F (−9.4 to −1.1 °C) according to the United States Department of Agriculture (USDA) Hardiness Zones [53].

We downloaded species occurrence data through the R package rgbif [54] for *C. pallida*, *O. tesota*, *P. florida*, and *P. microphylla* aggregated from the Global Biodiversity Information Facility (GBIF) [https://www.gbif.org/ (accessed on 21 March 2024)], an online collection of the data from various sources including museums, DNA barcodes, and community science platforms such as iNaturalist. Duplicated observations were removed based on geographical coordinates, date, dataset origin, and species identification number.

### 2.2. Climate and Elevation Data

Historical monthly climate data from 2000–2021 were obtained from WorldClim version 2.1 at a 2.5 min resolution (approximately 21 km^2^ at the equator) [https://worldclim.org/data/monthlywth.html (accessed on 14 December 2023)] [55,56]. The data contain the average minimum temperature (°C), average maximum temperature (°C) and average total precipitation (mm) for each month. The 19 bioclimatic variables (Table S3) that are generally used in species distribution models were averaged from the monthly data over the 21-year period. Two variables (bio3 and bio7) that were combinations of the other predictors were excluded from the models to reduce complexity and collinearity in the predictor dataset, which may result in a more accurate model [57,58,59].

Estimates of future (projected) climate data were obtained from AdaptWest for the 20-year period of 2021–2040 at a 30 s resolution [https://adaptwest.databasin.org/pages/adaptwest-climatena/ (accessed on 25 April 2024)], [60]. The data were resampled to attain a 2.5 min resolution to align with the 19 bioclimatic variables obtained from WorldClim. The projected monthly climate data is an ensemble of eight atmosphere-ocean coupled general circulation models (AOGCMs) from the Coupled Model Intercomparison Project Phase 6 (CMIP6) that have been noted by the Intergovernmental Panel on Climate Change (IPCC) as consistent with the most likely range of Earth’s equilibrium climate sensitivity [60,61,62].

For our study, we considered the intermediate Shared Socioeconomic Pathway (SSP) scenario of 2–4.5. This represents a future with increased global warming of 3 °C and additional radiative forcing of 4.5 W/m^2^ by the year 2100, given the current economic and developmental trends [4,63,64]. We also examined model projections for subsequent periods 2041–2060, 2061–2070, and 2081–2100 under SSP 2–4.5 and SSP 3–7.0 for each species. However, a comparison of all models showed no clear differences or additional insight than provided by SSP 2–4.5 in the near future (2021–2040) (Figures S1 and S2).

The 2023 Digital Elevation Model (DEM) data from the Commission for Environmental Cooperation (CEC) were downloaded directly from the site [http://www.cec.org/north-american-environmental-atlas/elevation-2023/ (accessed on 13 December 2023)]. The data depict North American terrain relative to mean sea level with a 250 m resolution, using data from the Global Multi-resolution Terrain Elevation Data (GMTED2010).

### 2.3. Species Distribution Model

Species distribution models (SDMs) for each species were constructed using MaxEnt (version 3.4.4) [57]. A model area specific to each species was determined by taking a minimum convex polygon of the respective occurrence points and extending that boundary by 150 km to account for species dispersal over time. The model uses the historical climate data from WorldClim and the elevation data from DEM for this study’s geographic region.

Occurrence data were thinned using the *gridSample* function (dismo package) to one observation per 2.5 min raster cell following the climate raster to reduce spatial autocorrelation and the effects of sampling bias [58,65]. This was performed for each species and applies to the remainder of the methods unless noted otherwise. The MaxEnt model was trained on 10,000 randomly selected pseudo-absence (a.k.a. background) points that were generated within the cropped model area using the *spatSample* function (terra package) [65,66,67].

Model tuning and evaluation were performed using the *ENMevaluate* function (ENMeval package) via the maxnet algorithm [68,69]. The k-fold method was used for partitioning the area into four spatial folds [70,71]. Three feature class combinations (L = linear, LQ = linear quadratic, and LQH = linear quadratic hinge) were selected alongside three regularization multipliers (1, 2, and 3), yielding 12 possible models for evaluation. We filtered model evaluation results by selecting one with an average Continuous Boyce Index (CBI) closest to a + 1, lowest average 10% omission rate, and highest average area under the curve (AUC) of the receiver operating characteristic (ROC) plot across all four folds [72,73,74,75].

### 2.4. Predicting Current and Future Distributions

Identifying potential suitable habitats for each species was performed by using parameter estimates from the aforementioned optimal model, in addition to environmental data raster stacks from the years 2000–2021 and 2021–2040. Raster stacks include the DEM and the respective time period being considered. Predictions were made using the *enm.maxnet@predict* function (ENMevaluate package), and the probability of presence was transformed by a complementary log-log [69,76]. Results with a 50% habitat suitability or greater are presented to highlight areas that are more likely to be suitable than unsuitable for each species.

## 3. Results

### 3.1. Most Occurrences Observed in Arizona and California (USA)

Collectively, a total of 13,214 observations of our focal species were gathered by community scientists across the southwest United States and northern Mexico and were used in this study (Table S1). Of the total number, 310 belonged to *C. pallida*, 4615 to *O. tesota*, 3769 to *P. florida*, and 4520 to *P. microphylla* (Table S1, Table S2). A majority of these observations were recorded in the United States, particularly in Arizona and California, and visually appeared to be grouped around more populated areas such as Phoenix, AZ and Tucson, AZ (Figure 1). The general distribution of *C. pallida* observations exhibited a roughly similar spread and area covered to that of all three of its host plants despite having only 7.5% as many observations as there were for each of the plants (Figure 1, Table S1).

### 3.2. Expansion in Predicted Suitable Habitat with a Shift to Higher Average Altitudes

**Figure 2:**
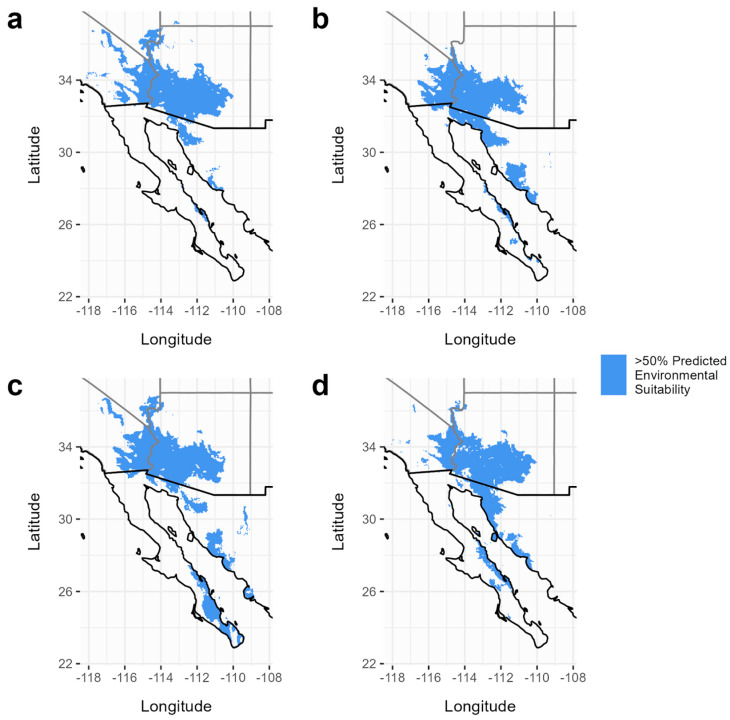
Species distribution models based on climate data from 2000–2021. Panels show the predicted distribution that has a greater than 50% environmental suitability for (**a**) *C. pallida*, (**b**) *O. tesota*, (**c**) *P. florida*, and (**d**) *P. microphylla*.

**Figure 3:**
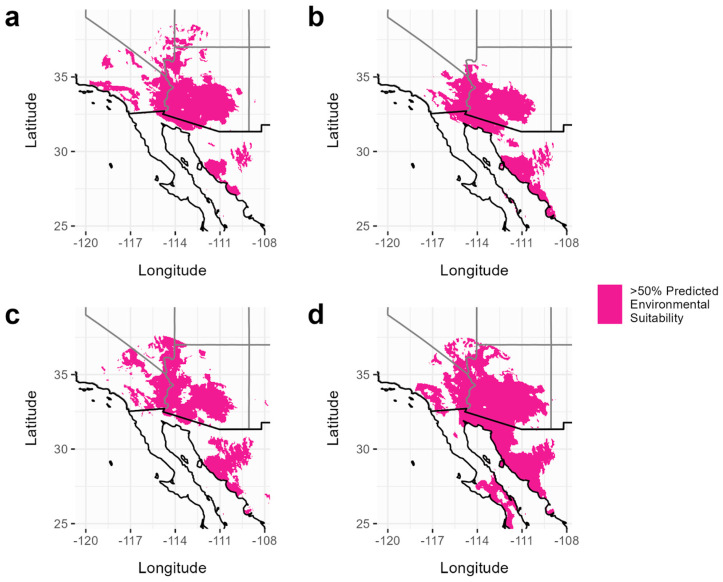
Forecasted species distribution models based on climate data from 2021–2040. Panels show the predicted distribution that has a greater than 50% environmental suitability for (**a**) *C. pallida*, (**b**) *O. tesota*, (**c**) *P. florida*, and (**d**) *P. microphylla*.

**Figure 4:**
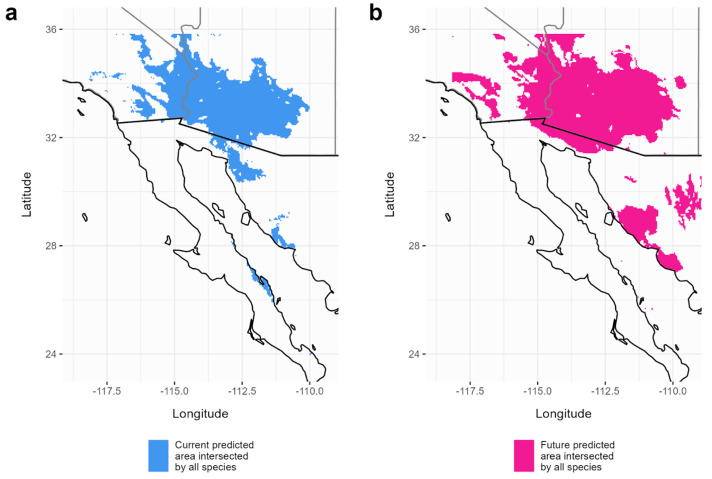
Forecasted changes in overlapped suitable areas. Panels represent the area where all species’ predicted environmental suitability (that is greater than 50%) intersects with each other, with (**a**) being the overlap in the current suitable area and (**b**) being that of the forecast (to the year 2040).

Here, we define suitable habitat as the areas identified to have a 50% or higher chance of being environmentally suitable for the species. The current predicted area suitable for *C. pallida* is estimated to be 163,307 km^2^, which lies within the range of the current predicted areas of its host plants (163,908–167,695 km^2^) (Figure 2, Table 1). Somewhat surprisingly, all species showed an expansion of habitat (with a greater than 50% suitability) between the two climate periods (2000–2021 and 2021–2040), ranging from an increase of 32% (*P. florida*) to 137% (*P. microphylla*) (Figure 3, Table 1). The forecasted distribution area of *C. pallida* continues to lie between the distribution areas of its host plants (229,231–396,604 km^2^) in the 2021–2040 period as well (Table 1). The current area of overlap between all four species is 150,851 km^2^, and this expanded to 215,759 km^2^ (a 70% increase) by the year 2040 under a model of moderate CO_2_ input (Figure 4). This increase in geographic area is accompanied by a shift to higher average elevations, with *C. pallida*’s average elevation increasing by 269 m and its host plants 93–272 m (Table 2), depending on species.

### 3.3. Focal Species’ Habitat Suitability May Be Linearly Related to Mean Temperature

With 17 bioclimatic variables in the best-fit models, we found that the mean temperature for the warmest quarter (bio10) was one of the top three contributors in predicting distributions for all four species (Table S3). Different combinations of variables contributed at varying capacities to each species’ model, but precipitation of the driest quarter (bio17) and precipitation of the coldest quarter (bio19) were utilized across all of the models (Table S3). The minimum temperature of the coldest month (bio6) was a top contributor in the models for the host plants (Table S3). Our focal desert species appear to benefit, at least to a small extent, from the projected warming temperatures (Figure 3).

The optimal model for *C. pallida* and *P. microphylla* was composed of a linear (L) feature and a regularization multiplier of 3 (Table S4). The other two species, *O. tesota* and *P. florida*, differed slightly with a linear quadratic (LQ) feature. The average values in our results suggest that the most fitting, but not overfit, models performed with a reasonable amount of accuracy. The best-fitting models for each species had an average AUC value between 0.71–0.81 (Table S4). The AUC value represents the proportion of correctly predicted observed absences, where 0 suggests poor predictive power, and 1 is the best [77]. The accompanying metrics, such as the average CBI values, ranging from 0.61–0.84, and the average 10% omission rates, indicated that the model predictions were also reasonably aligned with the distribution of the actual observations [73,75].

## 4. Discussion

Our results indicate a modest positive response of *C. pallida* to a slight increase in mean temperature (+1–2 °C) during the period of 2021–2040. We found the current suitable habitat for *C. pallida* to be 163,306 km^2^ (Figure 2), which is projected to expand by 59% to 258,869 km^2^ by the year 2040 (Figure 3, Table 1). Suitable habitat, defined here as areas with over a 50% chance of environmental suitability, for *C. pallida* that holds both abiotic conditions and all three host plants is currently predicted to be 150,851 km^2^ (Figure 4). High temperatures and aridity are most strongly associated with this expansion under the moderate climate scenario (SSP 2–4.5) (Figure 3, Table S3). Though even under a more severe emissions scenario (SSP 3–7.0) extending to 2100, all four of our desert species exhibited similar positive responses to the focal scenario (Figures S1 and S2). The overlap in suitable areas of *C. pallida* and its host plants is expected to increase by 64,908 km^2^, a 70% rise, primarily in northern regions (Figure 4). These findings resonate with existing studies, though limited, on arid-zone bees that also show minimal change in response to warming temperatures (e.g., [21,78]). These studies, conducted in the tropical dry forests of South America and the xeric regions of Australia, benefited from conducting an extensive sampling effort in addition to online databases for their species, likely enhancing their model output and accuracy. We were able to achieve similar and consistent results by solely utilizing GBIF data with a combination of museum collections and community science observations.

We found an average elevation increase for *C. pallida* by 269 m between the two time periods, with its host plants varying from 93 m to 272 m (Table 2). This predicted shift towards higher elevations aligns with meta-analyses indicating that species tend to move upslope in response to warming temperatures [79]. While some studies on animals or vegetation have documented elevational shifts upward towards cooler, moister climates or a downward shift in response to water availability from precipitation (e.g., [80,81,82,83]), the desert pallid bee may do so for physiological reasons. Previous research on *C. pallida* has shown that, like many animals, they tend to relocate to more favorable microhabitats to avoid reaching their critical thermal maxima, which ranges from ~111.2 °F (44.7 °C) (small males) to ~113 °F (46 °C) (females) [46,47,48]. Migrating to higher elevations would serve as an additional form for the bee to mitigate the effects of extreme climate (e.g., [78]). Regardless of direction, elevational and distributional shifts of desert vegetation and their pollinators are likely to induce shifts in associated species such as herbivores, parasitoids, and predators (e.g., [84,85]). Mainly, maintaining the overlap in distribution between pollinators and their host plants may contribute to sustaining ecological interactions and population persistence.

Species distribution modeling is an evolving approach with inherent limitations, yet it remains a valuable tool in ecological research (e.g., [36,37,38,86]). Our models reached acceptable evaluation metrics (Table S4), suggesting only minor changes in *C. pallida*’s range under different climatic scenarios (Figure S1). Depending on the species, the average AUC ranged from 0.71–0.81 and the average CBI from 0.61–0.84 (Table S4). Future studies could enhance model accuracy by incorporating additional factors such as land cover, surrounding vegetation, and soil type to better address the habitat needs of the desert pallid bee. The data in our study, sourced from GBIF, primarily consist of presence-only records from community scientists with varying levels of taxonomic expertise. Although GBIF is a popular open-source data hub frequently used for species distribution modeling (e.g., [87,88]), sampling biases remain a significant concern. Technologies such as computer vision are improving species identification accuracy and consistency [89,90]; however, sampling biases remain a concern now amplified with numerous participants. Most observers, extending back to museum collections in the 1950s, like to travel a maximum of two hours from their house and sample close to roads. Improved sampling in remote areas and including presence-absence observations could enhance the model output. However, to date, these types of comprehensive surveys remain much more costly compared to what most current biological monitoring resources can support.

With an eye toward the uncertainty of predicting many years into a complex and unknown future climate regime, we found that our four desert species in the arid southwest US and northern Mexico may be able to cope and persist with the forecasted climatic changes. Arizona, the center of the desert pallid bee distribution, is expected to experience higher average temperatures, wetter conditions in the north, and drier conditions in the south [91,92], which could create more suitable habitats at higher altitudes and northern regions for *C. pallida* as well as other lowland desert-adapted species. This is encouraging for maintaining ecosystem services such as pollination and plant recruitment necessary for soil stability and air quality in desert ecosystems. However, this interpretation may be negatively influenced by decreases in insect body size linked to increases in temperatures, which has been observed in *C. pallida* [47,93]. The decrease places further thermal stress on these bee populations as smaller insects are often less resilient to extreme heat [94,95]. The stability of the *C. pallida* alternative reproductive tactic (ART)—which likely relies on competing selective forces related to nesting density, female provisioning, and male mating success [96]—could be affected by declines in the species’ mean body size and the decreasing frequency of large-morph males. Similarly, the palo verde trees (*P. florida* and *P. microphylla*) may experience dieback at severe levels of drought and high evapotranspiration. Although desert species have adaptations to cope with high temperatures and arid conditions [14,15], the impacts on larval development are less clear, and it may be beneficial to explore the physiological limits of the bee and its host plants.

Broadly, our results suggest that desert species might be more resilient to warming compared to those in temperate and tropical regions [97,98], though species-specific responses will vary. Desert dwellers have evolved adaptations to withstand high temperatures and arid conditions, including efficient water use, heat dissipation, and microclimate utilization [14,15]. Combining these characteristics with potential trait plasticity and adaptation makes desert organisms more likely better prepared for future variations in climate extremes compared to their temperate and tropical counterparts [99].

In this study, we find that the desert pallid bee may expand its current range to new habitats and occupy more protected public land where conservation action is more feasible in North America, suggesting that immediate conservation action may not be necessary. However, species in regions experiencing more periods of extreme heat remain at risk despite their adaptations to arid climates [100,101]. Thus, while the expected geographic expansion and elevational shift involving *C. pallida* and its host plants may appear promising, proactive conservation management may still be necessary in the future as climate conditions evolve.

## 5. Conclusions

Our study found that the desert pallid bee (*C. pallida*) may tolerate and potentially benefit from warming temperatures (+1–2 °C) during the period of 2021–2040. Both *C. pallida* and its key host plants show a positive spatial relationship with rising temperatures, suggesting their distributions could expand slightly by 32% to 137% and shift to higher elevations in future years. The upward shift in elevation is consistent with existing research and implies that there may be physiological or behavioral limits to the predicted environmental conditions in lowland deserts. Overall, the positive response to a predicted warming climate suggests that desert bees may be more resilient to climate change compared to insects in tropical or temperate regions, offering a potential bright spot in insect conservation amidst widespread declines elsewhere.

## Figures and Tables

**Table 1:** Areas of current and forecasted suitable habitat (with a greater than 50% environmental suitability) in kilometers squared. The rightmost column shows the percent change between the two time periods.

Species	Current Distribution Area (km^2^)	Future Distribution Area (km^2^)	Percent Change (%)
*C. pallida*	163,307	258,869	59
*O. tesota*	163,908	229,231	40
*P. florida*	197,349	260,433	32
*P. microphylla*	167,695	396,604	137

**Table 2:** Average elevation across predicted suitable areas (with a greater than 50% environmental suitability) for current and future time periods. The rightmost column shows the change in elevation between the two time periods.

Species	Current Distribution Average Elevation (m)	Future Distribution Average Elevation (m)	Change in Elevation (m)
*C. pallida*	496	765	+269
*O. tesota*	329	422	+93
*P. florida*	390	662	+272
*P. microphylla*	448	663	+215

## Data Availability

All data acquisition, cleaning, analyses, and visualization were executed using the R Programming language [https://www.r-project.org/] (accessed on 13 December 2023), specifically through the RStudio environment version 2023.06.0+421 (“Mountain Hydrangea” Release for Windows) [https://posit.co/download/rstudio-desktop/] (accessed on 13 December 2023). The related code can be accessed at Zenodo [https://zenodo.org/doi/10.5281/zenodo.12539191] (accessed on 20 September 2024). The data and code used in this study are available at Github [https://github.com/Big-Biodiversity-Collaborative/DesertBees/tree/main] (accessed on 1 July 2024). These data were derived from the following resources available in the public domain: Global Biodiversity Information Facility [https://www.gbif.org/] (accessed on 21 March 2024), WorldClim 2.1 [https://worldclim.org/data/monthlywth.html] (accessed on 14 December 2023), Commission for Environmental Cooperation [http://www.cec.org/north-american-environmental-atlas/elevation-2023/] (accessed on 13 December 2023), and AdaptWest [https://adaptwest.databasin.org/pages/adaptwest-climatena/] (accessed on 25 April 2024). Additionally, the data and code have been archived at Zenodo [https://zenodo.org/doi/10.5281/zenodo.12539191] (accessed on 1 July 2024).

## Supplementary Materials

The following supporting information can be downloaded at https://www.mdpi.com/article/10.3390/insects15100793/s1, Figure S1: Forecasted SDMs for *C. pallida* based on climate data from three 20-year periods subsequent to 2021–2040, each under two carbon emission scenarios; Figure S2: Forecasted SDMs for the three host plants (*O. tesota*, *P. florida*, and *P. microphylla*) based on climate data from three 20-year periods subsequent to 2021–2040, each under two carbon emission scenarios; Table S1: Distribution of filtered observation numbers among states in the United States of America (USA) and Mexico (MX); Table S2: Number of records for each species used in the MaxEnt models; Table S3: Environmental variables included each species’ best-fit model and their percent contribution; Table S4: Best-fit model for each species.
