# Supplementary material for: Buzzing towards Resilience: Investigating the Spatial Alignment of the Desert Pallid Bee, Centris pallida, and Its Host Plants in Response to Climate Change
Source: Insects. 2024 Oct 11;15(10):793. doi: 10.3390/insects15100793 (PMC11508805; doi:10.3390/insects15100793)
Supplement: Supplementary file 1 [file insects-15-00793-s001.zip › insects-3239683-supplementary.pdf]

Supplementary Material

Figure S1. Forecasted species distribution models for *C. pallida* based on climate data from three 20-year periods subsequent to 2021-2040, each under two carbon emission scenarios. All panels show the predicted distribution that has a greater than 50% environmental suitability. Rows represent carbon emissions scenarios (a, b, c) SSP 2-4.5 and (d, e, f) SSP 3-7.0. Columns represent the periods (a, d) 2041-2060, (b, e) 2061-2080, and (c, f) 2081-2100.

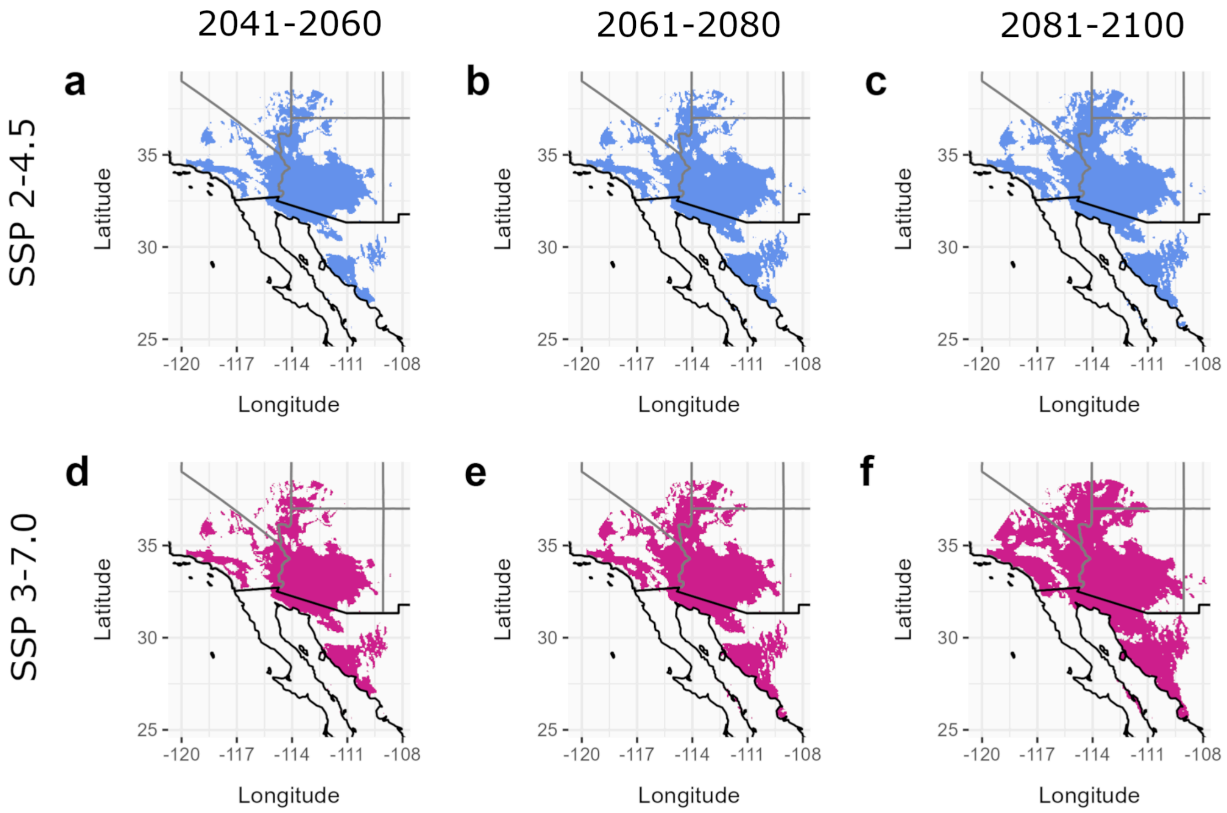

Figure S2. Forecasted species distribution models for the three host plants (*O. tesota*, *P. florida*, and *P. microphylla*) based on climate data from three 20-year periods subsequent to 2021-2040, each under two carbon emission scenarios. All panels show the predicted distribution that has a greater than 50% environmental suitability. Rows represent carbon emissions scenarios, SSP 2-4.5 and SSP 3-7.0, for each host plant. Columns represent the periods 2041-2060, 2061-2080, and 2081-2100, respectively.

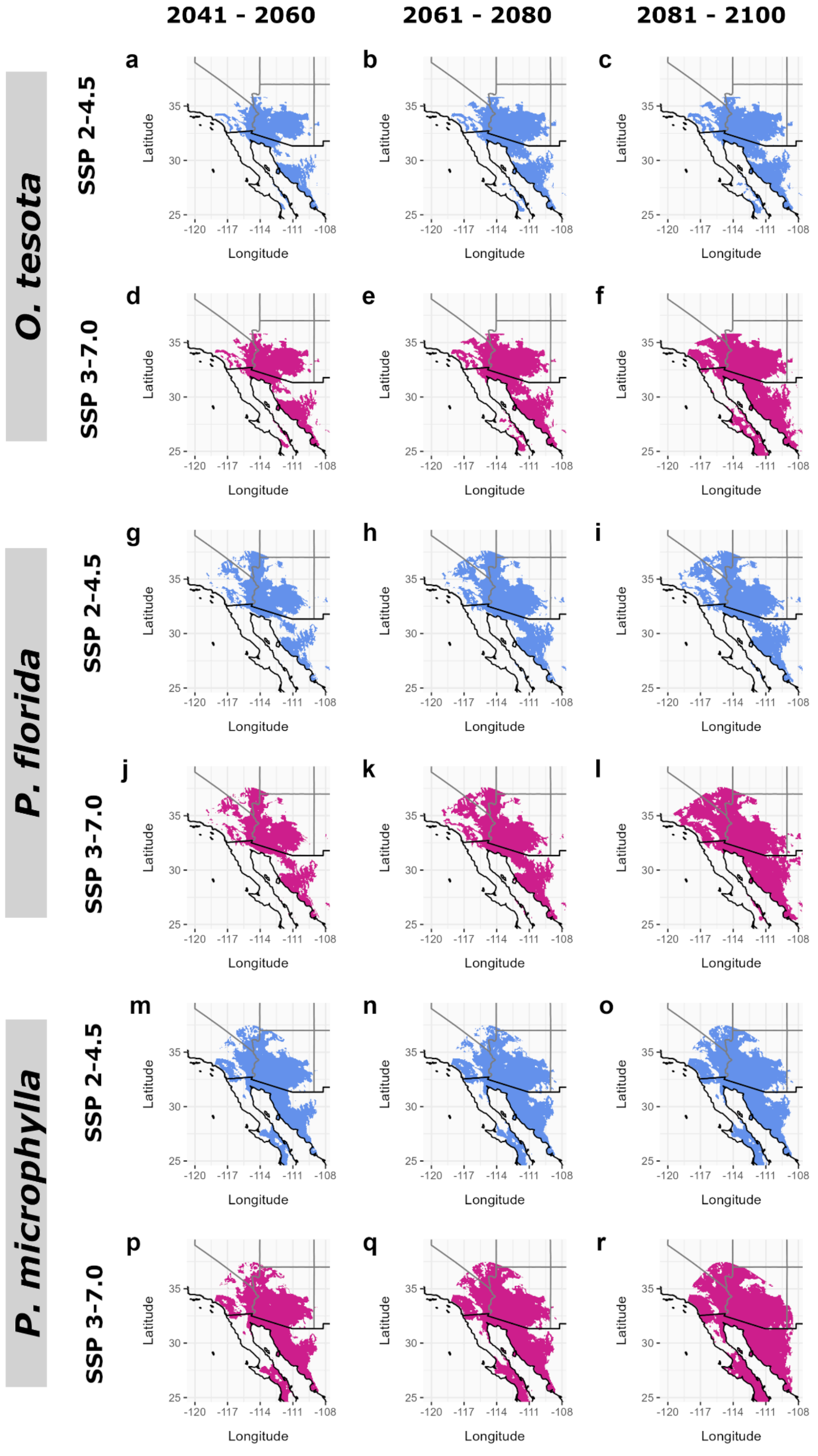

2041 - 2060
2061 - 2080
2081 - 2100
O. tesota
P. florida
P. microphylla
SSP 2-4.5
SSP 3-7.0
a
b
c
d
e
f
g
h
i
j
k
l
m
n
o
p
q
r
Latitude
Longitude
35
30
25
-120
-117
-114
-111
-108

Table S1. Distribution of filtered observation numbers among states in the United States of America (USA) and Mexico (MX).

**State / Country**	***# C. pallida***	***# O. tesota***	***# P. florida***	***# P. microphylla***
Arizona (USA)	235	2562	1605	3921
California (USA)	44	1372	1725	75
Nevada (USA)	17	0	11	2
New Mexico (USA)	1	0	1	1
Utah (USA)	2	0	0	0
Baja California (Norte and Sur) (MX)	8	340	282	326
Sinaloa (MX)	0	5	5	0
Sonora (MX)	3	336	140	195
**Total**	310	4615	3769	4520

Table S2. Number of records for each species used in the MaxEnt models. Data from GBIF was acquired for four species native to the Sonoran Desert. Observations from the earliest recording of each species, respectively, to 2023 were used. The total number of observations after cleaning is listed in the rightmost column, which includes observations that were omitted due to the distance from the known range.

**Species**	**Earliest Observation**	**Number of Records Used in Model**	**Number of Records Total**
C. pallida	1921	310	316
O. tesota	1882	4615	4619
P. florida	1846	3769	3785
P. microphylla	1882	4520	4528

Table S3. Environmental variables included each species' best fit model and their percent contribution. A bold asterisk (*****) beside a species' name in a variable row indicates that variable was the most positive contributor in habitat suitability predictions. Two (******) and three bold asterisks (*******) indicate the second and third most positive contributors, respectively. Variables in bold are those that were used in the model for all species: bio10 (mean temperature of warmest

quarter), bio15 (precipitation seasonality), bio16 (precipitation of wettest quarter), bio17 (precipitation of driest quarter), and bio19 (precipitation of coldest quarter).

**Variable**	**Description**	**Included in Model For**	**Percent Contribution (%)**
bio1	Annual Mean Temperature	*O. tesota* (***)	8.95
bio2	Mean Diurnal Range (Mean of monthly temperature range)	*C. pallida*<br>*P. microphylla*	-9.00<br>-1.72
bio3	Isothermality (bio2 / bio7) (×100)	Excluded from all models	0.00
bio4	Temperature Seasonality	*C. pallida*	0.10
bio5	Maximum Temperature of Warmest Month	*O. tesota*	0.63
bio6	Minimum Temperature of Coldest Month	*O. tesota* (*)<br>*P. florida* (*)<br>*P. microphylla* (**)	27.20<br>29.72<br>21.78
bio7	Annual Range of Temperature (bio5 - bio6)	Excluded from all models	0.00
bio8	Mean Temperature of Wettest Quarter	*C. pallida* (***)<br>*P. microphylla* (***)	6.51<br>11.47
bio9	Mean Temperature of Driest Quarter	*C. pallida*	0.08
**bio10**	**Mean Temperature of Warmest Quarter**	*C. pallida* (*)<br>*O. tesota* (**)<br>*P. florida* (***)<br>*P. microphylla* (*)	43.63<br>15.01<br>7.94<br>31.57
bio11	Mean Temperature of Coldest Quarter	None	0.00
bio12	Annual Precipitation	None	0.00
bio13	Precipitation of Wettest Month	None	0.00
bio14	Precipitation of Driest Month	*O. tesota*	8.78
bio15	Precipitation Seasonality	*C. pallida*<br>*O. tesota*<br>*P. microphylla*	2.57<br>0.38<br>0.04
bio16	Precipitation of Wettest Quarter	*C. pallida*<br>*P. microphylla*	-1.20<br>-1.22

**bio17**	**Precipitation of Driest Quarter**	*C. pallida* (**)<br>*O. tesota*<br>*P. florida* (**)<br>*P. microphylla*	8.62<br>6.81<br>11.81<br>6.16
bio18	Precipitation of Warmest Quarter	None	0.00
**bio19**	**Precipitation of Coldest Quarter**	*C. pallida*<br>*O. tesota*<br>*P. florida*<br>*P. microphylla*	2.85<br>0.95<br>0.26<br>2.90
elevation	2023 Digital Elevation Model of North America	*P. florida*<br>*P. microphylla*	0.04<br>0.11

Table S4. Best-fit model for each species. These were selected using the following criteria: (a) An average CBI closest to a +1, (b) lowest average 10% omission rate (OR), and (c) highest average AUC across all four folds.

Species	Feature class	Regularization multiplier	Avg. AUC	Avg. CBI	Avg. 10% OR	# of Coefficients
C. pallida	L	3	0.8058	0.7475	0.1360	9
O. tesota	LQ	3	0.7570	0.8445	0.0816	15
P. florida	LQ	3	0.7513	0.6148	0.097	13
P. microphylla	L	3	0.7118	0.8195	0.1568	9
